# A Bilocular Radicular Cyst in the Mandible with Tooth Structure Components Inside

**DOI:** 10.1155/2019/6245808

**Published:** 2019-09-03

**Authors:** Akari Noda, Masanobu Abe, Aya Shinozaki-Ushiku, Yae Ohata, Liang Zong, Takahiro Abe, Kazuto Hoshi

**Affiliations:** ^1^Department of Oral & Maxillofacial Surgery, University of Tokyo Hospital, Tokyo, Japan; ^2^Division for Health Service Promotion, University of Tokyo, Tokyo, Japan; ^3^Department of Pathology, Graduate School of Medicine, University of Tokyo, Tokyo, Japan; ^4^Department of Oral Pathology, Graduate School of Medical and Dental Sciences, Tokyo Medical and Dental University, Tokyo, Japan; ^5^Graduate School of Medicine, The University of Tokyo, Tokyo, Japan; ^6^Department of Gastrointestinal Surgery, Peking University Cancer Hospital & Institute, Beijing, China

## Abstract

**Background:**

A radicular cyst is the most common odontogenic cyst of inflammatory origin. Radiographically, it commonly demonstrates clear unilocular radiolucency; radicular cysts with multilocular radiolucency are quite rare.

**Case Presentation:**

A 64-year-old Japanese man who presented with a bilocular radiolucent lesion in his left mandible was referred by a dental clinic to our oral and maxillofacial surgery department. He had no particular subjective symptoms. Orthopantomography and computed tomography (CT) revealed an 18 mm × 15 mm lesion with well-defined bilocular radiolucency in the left mandible expanding from the distal side of a canine tooth to the bottom of the 2nd premolar. The lesion included the roots of the 1st and 2nd premolars. The root of the 2nd premolar showed knife-edge resorption. Although the 1st premolar was nonvital, the 2nd premolar was a vital tooth. As differential diagnoses, a radicular cyst, ameloblastoma, odontogenic keratocyst, pseudocyst, and others might be considered. We performed a total resection of the bilocular lesion and diagnosed the lesion as a radicular cyst with tooth structure components inside. The tooth structure components represented lamellar structures of cementum; they were located only in the proximal part (under the 1st premolar) of the lesion. The distal part of the lesion presented distinctive inflammation without tooth structure components.

**Conclusion:**

We encountered a rare case of a bilocular radicular cyst with tooth structure components inside.

## 1. Introduction

A radicular cyst is the most common odontogenic cyst of inflammatory origin [1]. It is commonly associated with pulpal necrosis leading to inflamed periapical tissues. Radiographically, a radicular cyst presents well-defined unilocular radiolucency. However, multilocular radiolucent radicular cysts are quite rare; there are few reported cases [2–4]. For multilocular radicular cysts, a differential diagnosis with other cysts and tumors (dentigerous cyst, ameloblastoma, odontogenic keratocyst, pseudocyst, etc.) is necessary [5]. Here, we describe the rare case of a bilocular radicular cyst located periapically to the first premolar of the fourth quadrant, presenting diagnostic difficulties.

## 2. Case Report

A 64-year-old Japanese man who presented with a bilocular radiolucent lesion in his left mandible was referred by a dental clinic to our department of oral and maxillofacial surgery. He had no subjective symptoms. His medical history included coronary vasospastic angina, diabetes mellitus, and hyperlipidemia.

**Figure 1:**
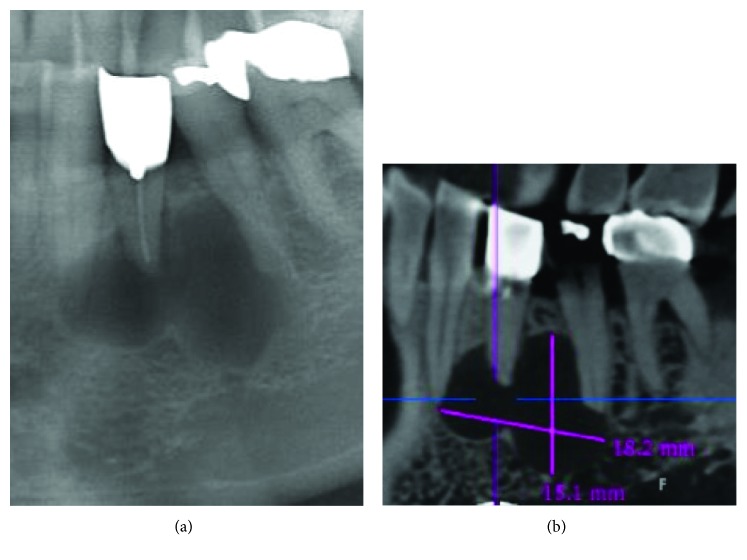
Clinical images of the bilocular lesion in the mandible. (a) Orthopantomography. The well-defined bilocular lesion is observed in the left mandible expanding from the distal of the canine tooth to the bottom of the 2nd premolar. (b) CT findings. The size of the bilocular lesion was 18 mm × 15 mm. The root of the 2nd premolar showed knife-edge resorption.

Orthopantomography and computed tomography (CT) revealed an 18 mm × 15 mm lesion with well-defined bilocular radiolucency in the left mandible expanding from the distal side of the canine tooth to the bottom of the 2nd premolar. The roots of the 1st and 2nd premolars were included in the lesion. Knife-edge root resorption was observed in the 2nd premolar (Figures 1(a) and 1(b)). Although the 1st premolar was nonvital, the 2nd premolar was found to be a vital tooth.

As a differential diagnosis, radicular cyst, ameloblastoma, odontogenic keratocyst, ameloblastic fibroma, odontogenic fibroma, odontogenic myxoma, jawbone central hemangioma, schwannoma, giant cell granuloma pseudocysts (simple bone cyst, aneurysmal bone cyst, and latent bone cyst), and hybrid lesions were considered.

**Figure 2:**
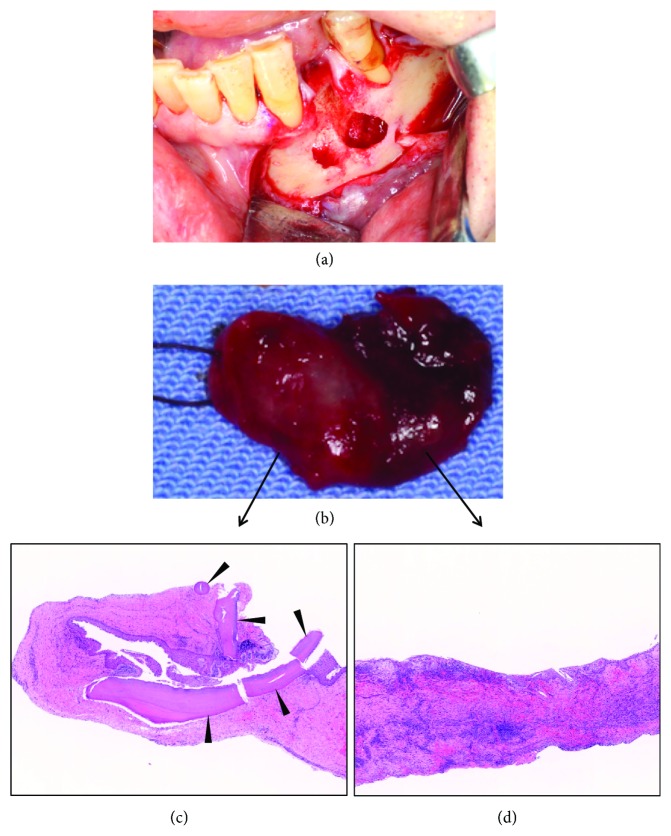
Intraoperative and pathological findings of the bilocular lesion in the mandible. (a) Bicameral bone resorption after resection of the bilocular lesion. After total resection of the bilocular lesion and extraction of the 1st premolar, bicameral alveolar bone resorption was observed. (b) The resected bilocular lesion. The left half is the proximal part of the lesion and the right side is the distal part. The proximal part is in bright red and the distal part is in dark red. (c) Pathological findings of the proximal part of the lesion. The components of hard tissue in the proximal part of the lesion represented lamellar structures of cementum (arrowheads). (d) Pathological findings of the distal part of the lesion, which showed severe inflammation without tooth structure components inside.

A biopsy was performed for the lesion under local anesthesia, because the knife-edge root resorption observed in the 2nd premolar suggested the possibility of ameloblastoma. When an ameloblastoma was identified in the biopsy, an adequate margin of safety was necessary for the resection. Based on the biopsy results, the lesion was diagnosed as a radicular cyst with a fibrous cystic wall and nonkeratinized squamous epithelium lining. However, there was a possibility that the incision biopsy did not reflect the entire lesion. We performed a total resection of the bilocular lesion with extraction of the 1st premolar under general anesthesia (Figures 2(a) and 2(b)). The inner surface of the entire lesion was covered by acanthotic squamous epithelium showing elongation of rete ridges. There was no clear border between the proximal and distal parts of the lesion.

The infiltration of inflammatory cells, plasma cells, and lymphocytes was extensive. Epithelial shedding as an effect of the inflammation was also observed (Figures 2(c) and 2(d)). Interestingly, tooth structure components with lamellar structures of cementum were found irregularly at the inner surface of the proximal part of the lesion (Figure 2(c)). The distal part showed severe inflammation without tooth structure components (Figure 2(d)).

## 3. Discussion

A radicular cyst is the most common odontogenic cyst, accounting for 55% of odontogenic cysts and 52%–68% of all of the cysts of the jaw in humans. Radicular cysts are observed mostly in the third and fourth decades of life, and they have shown male predilection. A “radicular cyst” is defined as a cyst arising from the epithelial residues in the periodontal ligament as a consequence of inflammation following necrosis of the dental pulp. The most common etiology is dental caries with pulp involvement [1, 2]. Radiographically, a radicular cyst presents well-defined unilocular radiolucency located periapical to a tooth with pulp involvement. In our patient's case, the radiographic appearance showed bilocular radiolucency apart from the usual radiolucency. There have been very few reports of a radicular cyst with multilocular radiolucency [2–4].

Before a pathological diagnosis, in addition to the radicular cyst, a differential diagnosis in similar cases should include ameloblastoma, odontogenic keratocyst, enamel epithelium fibroma, odontogenic fibroma, odontogenic myxoma, jawbone central hemangioma, schwannoma, giant cell granuloma, and pseudocysts (simple bone cyst, aneurysmal bone cyst, and latent bone cyst) [6–9]. We suspected ameloblastoma in the present case because knife-edge root resorption was observed in the 2nd premolar. The possibility of a hybrid lesion (cyst with cyst, cyst with tumor, and tumor with tumor) was also discussed, although such hybrids are uncommon [10–16]. Although dentigerous cysts associated with an adenomatoid odontogenic tumor have been reported in several cases, a radicular cyst associated with other cysts or tumors has not been reported, to the best of our knowledge [15, 16].

In our patient's case, tumors and pseudocysts were denied as the pathological diagnosis because the lesion had an apparent cystic wall. The whole resected tissue showed a stratified squamous epithelial lining. The wall of the lesion consisted of dense fibrous connective tissue, with an inflammatory infiltrate containing lymphocytes mixed with neutrophils, plasma cells, and histiocytes. These findings are in accordance with the pathology of a radicular cyst [17]. There was no clear border between the proximal and distal parts of the lesion. In only the proximal part of the lesion, tooth structure components were found irregularly at the inner surface of the cyst. The lesion was eventually considered a radicular cyst with tooth structure components that showed lamellar structures of cementum.

Although a radicular cyst with tooth structure components has not been reported, it is reasonable to speculate that the cementum-like structure in our patient's case was derived from odontogenic cells present in the wall that, under inflammatory stimulation, started to produce the structure over the years. As another hypothesis, we suspect that the cementum-like structure was derived from tooth fragments by the root canal treatments of the 1st premolar which had been performed several years before. The distal part of the lesion showed severe inflammation without tooth structure components. This suggested that a secondary infection that had occurred in the unilocular lesion caused severe inflammation which led to the formation of a bilocular cyst.

In summary, we experienced a rare case of a bilocular radicular cyst with tooth structure components inside. There are numerous reports about radicular cysts, but the multilocular type is quite uncommon. A radicular cyst with tooth structure components has apparently not been reported prior to this case.
